# Gene Expression Profiling of Liver Cancer Stem Cells by RNA-Sequencing

**DOI:** 10.1371/journal.pone.0037159

**Published:** 2012-05-14

**Authors:** David W. Y. Ho, Zhen Fan Yang, Kang Yi, Chi Tat Lam, Michael N. P. Ng, Wan Ching Yu, Joyce Lau, Timothy Wan, Xiaoqi Wang, Zhixiang Yan, Hang Liu, Yong Zhang, Sheung Tat Fan

**Affiliations:** 1 Department of Surgery, The University of Hong Kong, Pokfulam, Hong Kong, China; 2 Innovation Center China, AstraZeneca Global R&D, Shanghai, China; 3 Beijing Genomics Institute (BGI), Shenzhen, China; 4 State Key Laboratory for Liver Research, The University of Hong Kong, Pokfulam, Hong Kong, China; The University of Texas MD Anderson Cancer Center, United States of America

## Abstract

**Background:**

Accumulating evidence supports that tumor growth and cancer relapse are driven by cancer stem cells. Our previous work has demonstrated the existence of CD90^+^ liver cancer stem cells (CSCs) in hepatocellular carcinoma (HCC). Nevertheless, the characteristics of these cells are still poorly understood. In this study, we employed a more sensitive RNA-sequencing (RNA-Seq) to compare the gene expression profiling of CD90^+^ cells sorted from tumor (CD90^+^CSCs) with parallel non-tumorous liver tissues (CD90^+^NTSCs) and elucidate the roles of putative target genes in hepatocarcinogenesis.

**Methodology/Principal Findings:**

CD90^+^ cells were sorted respectively from tumor and adjacent non-tumorous human liver tissues using fluorescence-activated cell sorting. The amplified RNAs of CD90^+^ cells from 3 HCC patients were subjected to RNA-Seq analysis. A differential gene expression profile was established between CD90^+^CSCs and CD90^+^NTSCs, and validated by quantitative real-time PCR (qRT-PCR) on the same set of amplified RNAs, and further confirmed in an independent cohort of 12 HCC patients. Five hundred genes were differentially expressed (119 up-regulated and 381 down-regulated genes) between CD90^+^CSCs and CD90^+^NTSCs. Gene ontology analysis indicated that the over-expressed genes in CD90^+^CSCs were associated with inflammation, drug resistance and lipid metabolism. Among the differentially expressed genes, glypican-3 (GPC3), a member of glypican family, was markedly elevated in CD90^+^CSCs compared to CD90^+^NTSCs. Immunohistochemistry demonstrated that GPC3 was highly expressed in forty-two human liver tumor tissues but absent in adjacent non-tumorous liver tissues. Flow cytometry indicated that GPC3 was highly expressed in liver CD90^+^CSCs and mature cancer cells in liver cancer cell lines and human liver tumor tissues. Furthermore, GPC3 expression was positively correlated with the number of CD90^+^CSCs in liver tumor tissues.

**Conclusions/Significance:**

The identified genes, such as GPC3 that are distinctly expressed in liver CD90^+^CSCs, may be promising gene candidates for HCC therapy without inducing damages to normal liver stem cells.

## Introduction

Hepatocellular carcinoma (HCC) is the fifth most common cancer in the world with a high mortality rate [1]. Most HCC patients present at an advanced stage, which is refractory to chemotherapy and radiotherapy [2], [3]. Moreover, the recurrence rate of this disease is very high after curative treatment [4]. Understanding the mechanism of carcinogenesis is pivotal for the management of HCC [5].

Lines of evidence have revealed the existence and importance of cancer stem cells (CSCs) in carcinogenesis in the past decades. CSCs are considered to be the root of cancers, and are responsible for tumor growth and differentiation of heterogeneous cell populations within tumors [6]. Additionally, they have been demonstrated to be chemoresistant [7] and radioresistant [8]. In our previous study, using the surface marker CD90 (Thy-1, expressed by hepatic stem/progenitor cells), liver CSCs were identified in HCC cell lines, tumor specimens and peripheral blood samples of HCC patients and these CD90^+^CSCs displayed tumorigenic capacity [9].

Since the capacities of tumorigenicity, differentiation, self-renewal and chemoresistance of liver CD90^+^CSCs are governed by their distinctive genetic makeup and an array of gene expression changes in biological processes, elucidation of their molecular profile is important in understanding the characteristics of these cells. Nevertheless, comprehensive gene expression profiling of liver CSCs remains to be determined.

In the past decades, cDNA microarray has been extensively used to identify differential gene expression profiles in many cancers for screening, prognosis and tumor classifications [10]–[13]. However, cDNA microarray suffers from intrinsic limitations, such as low sensitivity, low dynamic ranges [14], and hybridization artifacts [15]. In fact, the majority of genes in biological processes, such as those encoding transcription factors and signal transducers, usually express at low levels [16]. Hence, cDNA microarray may not be an ideal tool to delineate molecular pathways. In this study, we used next-generation RNA sequencing (RNA-Seq), taking advantage of its superior sensitivity and capability of detecting splice variants, to sequence the whole transcriptomes of liver CD90^+^CSCs and CD90^+^ non-tumorous stem cells (NTSCs) from three HCC patients. The differential expression of genes was examined between these two groups of CD90^+^ cells and the results were validated by quantitative reverse transcriptase polymerase chain reaction (qRT-PCR). Concordant results were indicated between the platforms of RNA-Seq and qRT-PCR, and a majority of transcripts were detected at low expression levels by RNA-Seq. Besides, more structural isoforms were found in liver CD90^+^CSCs than CD90^+^NTSCs. Further, Gene Ontology (GO) analysis indicated that the up-regulated genes were associated with drug metabolism, lipid metabolism and inflammation which may account for drug resistance, cell proliferation, and progression of the tumor. Among the up-regulated genes identified, Glypican-3 (GPC3), a member of glypican family of heparan sulfate proteoglycans, was over-expressed in CD90^+^CSCs. By immunohistochemical staining, GPC3 was detected in the majority of liver tumor tissues, but absent in adjacent non-tumorous tissues. Interestingly, the GPC3 expression level was positively correlated to the number of CD90^+^CSCs in liver tumor tissues. Further investigation by flow cytometry indicated that GPC3 was remarkably expressed in CD90^+^CSCs in human liver tumor specimens. Based on our current findings, regardless of ambiguous roles of GPC3 on liver cancer stem cells, GPC3 could be a promising target gene for HCC immunotherapy owing to its specificity on the liver cancer stem cells, and its absence in normal liver stem cells.

## Materials and Methods

### Patients and sample collection

All patients signed a written informed consent, and the data and samples were analyzed anonymously. The present study was approved by the Institutional Review Board of The University of Hong Kong. A total of 15 patients were recruited for RNA-Sequencing and validation in this study (Table 1). The mean age of these patients was 57. There were 14 men and 1 woman. Thirteen of these patients were positive for serum hepatitis B surface antigen and 1 for hepatitis C antibody. Eighty-seven percent of these patients presented at tumor-node-metastasis (TNM) stage III or IV with a mean tumor size of 9.3 cm. The three HCC patients whose specimens were studied by RNA-Seq were male, aged from 55 to 61. All were hepatitis B virus carriers. Two had TNM stage III cancer and one had TNM stage II cancer. Pathological diagnosis was made according to the histology of tumor specimens examined by experienced pathologists.

**Table 1:** Clinicopathological features of HCC patients used for RNA-Seq analysis and the prospective validation of RNA-Seq analysis by qRT-PCR.

Clinicopathological details of patients (N = 15)	Frequency
**Mean Age (Range)**	57(41–83)
**Sex**	
**Male**	14
**Female**	1
**HBV**	13
**HCV**	1
**TNM stage**	
**I**	0
**II**	2
**III**	8
**IV**	5
**Mean Tumor Size (Range)**	9.3 cm (2–18)
**Mean Serum AFP (Range)**	17,244 ng/ml (3–211,427 )

The three HCC patients whose specimens were studied by RNA-Seq were male, aged from 55–61. All were HBV carriers. Two had tumor TNM stage III and one had tumor TNM stage II.

Tumor and parallel non-tumorous liver tissues were harvested at the time of operation. The cell isolation procedure from liver tissues was performed as previously described with some modifications [9]. In brief, after digestion with 100 units/ml type IV collagenase (Sigma-Aldrich, St Louis, MO, USA) for 30 minutes at 37°C, tissues were minced and cell suspension was passed through a 100-µm nylon mesh to remove tissue debris. Red blood cells (RBC) were then lysed by RBC lysis buffer and the cell suspension was washed again, finally passed through a 40-µm nylon mesh. Cells were resuspended with buffer and HetaSep (Stem Cell Technology, Vancouver, BC, Canada) was then added into the cell suspension for removal of remaining debris. After dead cell removal, the cells were eventually resuspended in staining buffer (2% BSA, 2 mM EDTA in PBS), counted and subjected to flow cytometry analysis and cell sorting. Cells were also sorted onto glass slide, counterstained with DAPI and examined under fluorescence microscope.

### Cell lines

PLC and MHCC97L cell lines [9] were maintained as monolayer culture in high glucose DMEM with 10% fetal bovine serum and 1% penicillin/streptomycin (Life Technologies, Carlsbad, CA, USA) at 37°C in a humidified atmosphere of 5% CO_2_ in air.

### Fluorescence-activated cell sorting (FACS) for CD90^+^ cells

The isolated cells from tumor and non-tumor tissues were labeled with PE-conjugated anti-human CD90 and APC-conjugated anti-human CD45 antibodies (BD Pharmingen, San Diego, CA, USA). Subsequently, CD45^−^CD90^+^ cells were isolated using a BD FACSAria II Cell Sorter (Becton Dickinson Immunocytometry Systems, San Jose, CA, USA). An aliquot of CD90^+^ cells were checked for purity. The isolated cells were further treated with RNA™safer RNA stabilization reagent (SABiosciences, Frederick, MA, USA) and the cell pellets were stored at −80°C for subsequent RNA isolation.

### Flow Cytometry analysis of HCC cell lines and human liver tumor tissues for GPC3 and CD90

Initially viable PLC and MHCC97L cells and viable cells from human liver tumor tissues after digestion were sorted using Sytox Blue (Invitrogen) according to the manufacturer's instruction, then the cells were fixed and permeabilized with the fixation/permeabilization kit (BD Biosciences, San Diego, CA, USA). After washing, the cells were stained with a PE-conjugated anti-CD90 (BD Pharmingen, San Diego, CA, USA) and an anti-GPC3 antibody (Santa Cruz Biotechnology, Santa Cruz, CA, USA) labeled with Zenon® Alexa Fluor® 488 mouse IgG1 Labeling Kit (Life Technologies, Grand Island, NY, USA). Following incubation and washing, the stained cells were detected and counted by a BD FACSAria II (Becton Dickinson Immunocytometry Systems, San Jose, CA). Appropriate isotypes were used as controls.

### RNA isolation and RNA amplification

Total cellular RNAs were extracted from the isolated CD45^−^CD90^+^ cell pellet using an RNAqueous-Micro RNA isolation kit (Ambion, Austin, TX, USA). The RNA samples (∼50 ng) were then amplified with a MessageAmp II aRNA Amplification kit (Ambion) according to the manufacturer's instruction. In brief, a double stranded cDNA was synthesized by reverse transcription from RNA with a T7 promotor primer. After purification, the double-stranded cDNA acted as a template for in vitro transcription to generate multiple copies of amplified RNA (aRNA). Following RNA amplification, the aRNA was subjected to a second round of amplification with the same methodology as the first amplification except using a different primer provided by the manufacturer. A control Hela RNA was also run in parallel with the RNA samples during the amplification procedure. After completion of amplification, the concentration of aRNA was measured using Nanodrop ND-1000 and the quality of the aRNA was analyzed by the Bioanalyzer 2100 (Agilent Technologies, Santa Clara, CA, USA).

### RNA library preparation and sequencing

RNA-library preparation was performed according to the manufacturer's recommendations. In brief, the poly-A containing aRNAs were purified, followed by fragmentation of RNA into small pieces. The cleaved RNA fragments were synthesized into single-strand cDNA using superscript II reverse transcriptase (Invitrogen) and random hexa-primers (IDT, Coralville, Iowa, USA), followed by second strand synthesis with DNA polymerase I (Invitrogen) and E. coli RNase H (Invitrogen). After second strand synthesis, with end repair and A-tailing, the synthesized double-stranded cDNA fragments were subjected to purification, then ligated to Illumina adapters using Quick ligation TM kit (NEB) and DNA ligase. The resultant cDNA adapter-modified cDNA libraries were fractionated on agarose gel, 200-bp fragments were excised and amplified by 15 cycles of polymerase chain reaction. After purification, the quality of cDNA libraries was checked by Bioanalyzer 2100 (Agilent). The concentration of cDNA libraries was measured and diluted to 10 nM in Tris-HCl buffer prior to cluster generation. Cluster formation, primer hybridization and sequencing reactions were performed sequentially according to the manufacturer's recommended protocol. In the present study, we used pair-end sequencing by Illumina Genome Analyzer II (Illumina, San Diego, CA, USA) with 76 cycles. One lane of flow cell was used for each sample. Raw short sequence fragments were accepted if they passed the quality filtering parameters used in the Illumina GA Pipeline GERALD stage.

### Read mapping and gene expression

High-quality reads were aligned to the human reference genome (NCBI Build 36.1) using NextGENe® software (Softgenetics, State College, PA, USA). The matched reads were aligned to Human Refseq mRNA (NCBI). Reads shorter than 20 bps and those with the quality score less than 14 were excluded. The sequences aligned with individual transcript were counted digitally. The expression levels for each gene were normalized to reads per kilobase of exon model per million mapped reads (RPKM) to facilitate the comparison of transcripts among samples. Tophat software was used to identify splice variants of each sample [17].

### RNA-Seq data mining and defining mis-regulated genes across the patients

A large database containing all gene transcripts identified by RNA-Seq for the samples of CD90^+^ cells from paired tumor and non-tumorous tissues of 3 patients were assembled. A mean log_2_ fold change [RPKM of CD90^+^CSCs/RPKM of CD90^+^NTSCs] of each gene was calculated across all 3 patients. The false discovery rate (FDR, i.e. a probability of wrongly accepting a difference between these two tested CD90^+^ cell groups) of each gene was determined according to Storey's method [18]. The genes were regarded as differentially expressed when their FDRs were less than 0.05. Further, genes were classified as up-regulated when their mean log_2_ fold change ratio was larger than 1 or down-regulated when their log_2_ fold change ratio was less than −1.

### Fluidigm microfluidic chips for qRT-PCR

To validate the reliability of RNA-Seq data, initially we performed qRT-PCR using the same amplified RNA materials as an internal validation, followed by original, non-amplified RNA from an independent HCC patient cohort as a prospective validation. BioMark® Real-Time PCR System 48.48 Dynamic Array (Fluidigm, South San Francisco, CA, USA) was used to perform the qRT-PCR according to the manufacturer's protocols.

EvaGreen and TaqMan assays were done according to the manufacturer's protocols. The primers of selected genes were designed using Primer 3 software (Table S1). TaqMan Universal Master Mix, TaqMan PreAmp Master Mix and probes were purchased from Applied Biosystems (ABI, Foster City, CA, USA). Samples were run at least in duplicate. The gene expression level was normalized by subtracting the cycle threshold (Ct) of an abundantly-expressed control gene from the Ct for each selected gene of interest. Relative gene expression values expressed as fold change were subsequently determined using the 2^−ΔΔCT^ method. GAPDH was used as the reference control gene and the CD90^+^NTSCs were taken as the reference samples. Data were analyzed using the BioMark Real-Time PCR Analysis Software version 2 (Fluidigm).

The quantification of gene expression of GPC3 was performed by Fluidigm Digital Array. The assay was performed according to the manufacturer's protocol and the data were analyzed by the use of BioMark Digital PCR Analysis software (Fluidigm).

### Quantification of CD90^+^ cells in human liver tumor samples by flow cytometry

Tumor and parallel non-tumorous liver tissues were obtained from another forty-two HCC patients at the time of hepatectomy. A portion of each resected tissues was fixed in 10% formalin and embedded in paraffin. The remaining portion underwent the same procedures of FACS for CD90^+^ cells as described above and the number of CD90^+^ cells were analyzed and quantified by BD FACSCalibur flow cytometer (Becton Dickinson Immunocytometry Systems).

### Immunohistochemical staining of GPC3 in human liver tumor samples

The embedded tissues were cut into 5-µm thick sections for immunohistochemical staining of GPC3. The sections were initially deparaffinized in xylene and rehydrated through ethanol to water. The sections were then treated with 3% hydrogen peroxide in methanol for 20 minutes to abolish endogenous peroxidase activity. For antigen retrieval, sections were heated in 10 mM citrate buffer (pH 6.0) with pressure cooker for 5 minutes at 120°C. The sections were subsequently covered with a 1∶1000 dilution of mouse anti-GPC3 monoclonal antibody in PBS (Santa Cruz Biotechnology, California, USA) for 1 hour at room temperature. After washing with TBS-Tween 20, the sections were incubated with envision Polymer-horseradish peroxidase (DakoCytomation, Carpenteria, CA, USA), which was used as a secondary antibody for 30 minutes at room temperature. The color signal for GPC3 of each section was developed by the addition of 3, 3 diaminobenzidene tetrahydrochloride (DakoCytomation), followed by 2 minute incubation. Finally the sections were washed with distilled water and counterstained for nuclei with 10% hematoxylin and dehydrated. The analysis of immunohistochemistry was performed by independent researchers. The GPC3 expression was assessed using an “H score” system, which was obtained by the following formula:

3X percentage of strong staining+2X percentage of moderate staining+percentage of weak staining [19].

### Small Interfering RNA (siRNA) transfection in vitro

A specific GPC3-siRNA and a scrambled siRNA control (SSC) were purchased from Ambion (Austin, TX). CD90^+^GPC3^+^ cells were sorted from PLC cells for subsequent functional assays, as PLC cells express relatively high CD90 and GPC3. GPC3 knockdown was achieved by transfecting siRNA oligo into the sorted cells using the reverse transfection with Lipofectamine RNAiMAX reagent (Invitrogen) according to the manufacturer's instructions at a final concentration of 20 nM siRNA. These transfected cells are henceforth annotated as PLC CD90^+^GPC3^+(GPC3−)^. In parallel, PLC CD90^+^GPC3^+^cells were transfected with the scrambled siRNA control at a final concentration of 20 nM, which are henceforth annotated as PLC CD90^+^GPC3^+^^(ssc)^.

### Cell proliferation Assay

PLC CD90^+^GPC3^+(GPC3−)^ and CD90^+^GPC3^+^^(ssc)^ cells were seeded onto a 96-well plate at a density of 4,000 cell/well in DMEM/10% FBS. At the indicated time points, 10 ul of WST-1 reagent (Roche Applied Science, Madison, WI, USA) was added into each well containing 100 ul medium. The plate was incubated for 2 hours, followed with 1 minute shaking. Cell growth was assessed by measuring absorbance at 450 nm using a microplate reader (Thermo Fisher Scientific, Waltham, MA, USA ) on day 1, 2, 3 and 4. Each sample was run in triplicate and expressed as mean±SD. At least two independent experiments were performed.

### Stem cell colony formation assay

Clonogenic capacity of cancer stem cells was assessed by stem cell colony formation assay. The CD90^+^GPC3^+^ cells were isolated from PLC cells, followed by transfection with GPC3 siRNA and scrambled siRNA control, respectively. The CD90^+^GPC3^+(GPC3−)^ and CD90^+^GPC3^+^^(ssc)^ were seeded into semisolid agar media using StemTAG TM 96-well stem cell colony formation assay kit (Cell Biolabs, Inc. San Diego, CA, USA) according to the manufacturer's instruction. In brief, 50 ul of Base Agar Matrix Layer was dispensed into each well of a 96-well plate and solidified at 4°C. Seventy-five ul of cell suspension/agar matrix suspension containing 5,000 cells was dispensed into each well. After solidifying, 50 ul of culture medium with growth factors was added into each well and the cells were incubated for 8 day in the humidified incubator at 37°C with 5% CO_2_. The colony formation ability was examined under a microscope, and the results were then determined by quantifying alkaline phosphatase activity after cell lysis.

### Statistical analysis

The continuous variables were expressed as mean ± S.D or median. Comparisons of the fold change of genes and splice variants between two groups were performed by Student's t-test. Correlation between measured genes between RNA-Seq and qRT-PCR was measured by Spearman rank correlation coefficient. All analyses were performed with the GraphPad Prism 5 software (GraphPad Software, La Jolla, CA, USA). A *P* value less than 0.05 was considered statistically significant.

## Results

### Isolation of CD90^+^ cells from tumor and non-tumor specimens

Both anti-human CD90 and anti-human CD45 antibodies were used in the isolation procedures. As CD90 was also expressed by some lymphocytes, a combination of CD45^−^CD90^+^ was used to define nonlymphatic CD90^+^ cells in the patient liver tissues (Figure S1). The BD cell sorter sorted CD90^+^ cells with an average purity of 86.6%. A median number of 3.4×10^4^ CD90^+^CSCs from tumor tissues and 8.9×10^3^ cells of CD90^+^NTSCs from non-tumorous tissues were obtained. The number of CD90^+^CSCs was significantly higher than that of CD90^+^NTSCs (*P* = 0.0008). The sorted CD90^+^cells were further confirmed by immunofluorescence staining before RNA extraction.

### RNA extraction and amplification

The yield of RNA from the sorted CD90^+^ cells ranged from 12 ng to 200 ng (from 10^3^ to 10^4^ cells). Owing to the insufficient amount of RNA for RNA-Seq, two rounds of RNA amplification were performed according to the manufacturer's procedures. After amplification, an average of 114 µg of amplified RNA (aRNA) was obtained. The sizes were in the range of 200–2000 nucleotides, with the majority being around 500, which is in concordance with the specifications of the sizes of aRNA stated by the manufacturer, indicating that the aRNA samples were in good quality.

Regarding the bias of RNA amplification in the RNA-Seq technology, no comparison data has ever been found in the literature yet. Nevertheless, it has been demonstrated that one or two rounds of RNA amplification generated reproducible microarray data without significant loss of gene detection [20]. Additionally, a study showed that 75% more genes were detected by mRNA sequencing compared to microarray after cDNA amplification on a single cell [21]. Hence, we believed that the produced aRNAs could also be used for RNA-Seq.

### Sequencing-by-synthesis of amplified RNA isolated from CD90^+^CSCs and CD90^+^NTSCs on Illumina Genome Analyzer II

The amplified RNAs after construction of a cDNA library were subjected to RNA-Seq on Illumina Genome Analyzer II (pair-end sequencing). On completion, 14.9 million to 20.5 million 75-bp long sequence reads per sample were generated, and they corresponded to an average of 1.30 Gb raw sequence data. Alignment to mRNA Reference Sequence (NCBI) was 39.4±7% with a greater portion of reads aligned to the reference human genome (70.3±8%) (Table 2), suggesting that the unaligned sequences to RefSeq was probably due to incomplete annotation of mRNA isoforms in Homo sapiens [22], [23]. Other causes might be attributed to their origins outside the reference human genome or low sequence quality [24]. Nevertheless, the average number of transcripts detected in CD90^+^CSCs and CD90^+^NTSCs were 31,407±2,202, and 31,444±479, respectively, which corresponded to about 74% of Human RefSeq transcripts entries. Because no significant difference in the number of transcripts was found between the two types of CD90^+^ cells, the gene comparison was considered to be valid (*P* = 0.7).

**Table 2:** Alignment statistics for transcriptome reads of CD90^+^ cells isolated from tumor and non-tumor tissues from 3 HCC patients.

	Patient A		Patient B		Patient C	
	CD90^+^CSCs	CD90^+^NTSCs	CD90^+^CSCs	CD90^+^NTSCs	CD90^+^CSCs	CD90^+^NTSCs
**Total Reads Processed**	19.1 M (100%)	20.5 M (100%)	16.4 M (100%)	16.1 M (100%)	14.9 M (100%)	17 M (100%)
**Reads matched to Ref RNA**	7.7 M (40.3%)	10 M (48.7%)	5.3 M (32.3%)	5.02 M (31.1%)	5.5 M (37%)	8.0 M (47.2%)
**Reads matched to Ref Genome**	14.8 M (77.6%)	14.7 M (71.8%)	9.8 M (60%)	9.8 M (61%)	10.9 M (73.3)	13.3 M (78.1%)
**Transcript matches (1000 s)**	33.4	31.3	32.4	32.2	29.2	31.5
**Average transcript coverage**	18×	28×	16×	15×	18×	24×

Reads counts are expressed in million or a percentage of the total reads processed for each sample in parentheses.

Alternative precursor messenger RNA (pre-mRNA) splicing plays important roles in the generation of functional diversity of the genome. Accumulating evidence has revealed that aberrant splicing contributes to neoplasia, cancer progression and metastasis [25]–[27]. Splicing events including alternative 3′ or 5′ splice site, alternative first exon, alternative last exon, intron retention, and exon skipping were compared between CD90^+^CSCs and CD90^+^NTSCs (Table 3). The average numbers of alternative splicing of CD90^+^CSCs were found to be 383, whereas those of CD90^+^NTSCs were 245 (*P*<0.05). More structural variants were found in CD90^+^CSCs as compared to CD90^+^NTSCs, indicating that more regulatory and functional diversity of transcriptomes occurred in liver CSCs.

**Table 3:** Alternative splicing of CD90^+^CSCs and CD90^+^ NTSCs.

	CD90^+^CSCs	CD90^+^NTSCs
**Alternative 3′ splice site**	127	77
**Alternative 5′ splice site**	115	74
**Alternative first exon**	23	15
**Alternative last exon**	11	11
**Mutually exclusive exon**	1.7	0.3
**Intron retention**	44	23
**Exon skipping**	61	45
**Total number of alternative splicing events**	383	245

Events are expressed as means. More alternative splicing events were observed in CD90^+^CSCs as compared with CD90^+^NTSCs (*P*<0.05).

**Figure 1:**
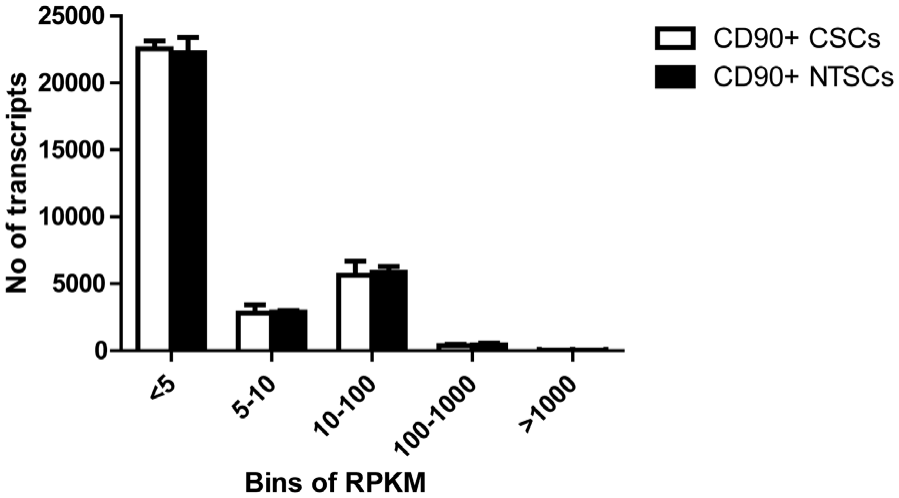
Bar chart showing the number of reads at different levels. Y-axis, number of reads; X-axis, bins of expression levels (bins at <5 RPKM, 5–10 RPKM, 11–100 RPKM, 100–1000 RPKM and >1000 RPKM). The majority of the transcripts were expressed at low levels (<5 RPKM). RPKM, reads per kilobase per million of reads.

The distribution of transcripts in CD90^+^CSCs and CD90^+^NTSCs exhibited similar patterns (Figure 1). We observed that 80% of transcripts had less than 10 RPKM in CD90^+^CSCs or CD90^+^NTSCs, but only about 0.1% of the expressed transcripts had more than 1000 RPKM. This implied that the majority of transcripts were expressed at low levels and might not be easily identified by the cDNA microarray. A similar transcript distribution pattern was found in the study of gene expression profiling in glioblastoma using the next-generation sequencing technology [28]. This again demonstrates high sensitivity of RNA-Seq in detecting lowly expressed transcripts in cancer cells [29].

### Analysis of gene expression profiles of CD90^+^CSCs and CD90^+^NTSCs

Upon removal of duplicate genes after aligning transcripts to the Human RNA Reference Sequence, the gene expression profiles of 24,609 genes were analyzed. Five hundred genes were differentially expressed, among which 119 genes were up-regulated, and 381 were down-regulated.

In the previous and present studies, we used anti-human CD90 antibody to isolate CD90^+^CSCs and CD90^+^NTSCs from patient tumor tissues because CD90 is a surface marker for hepatic stem cells (oval cells) [30]. These two groups of cells exhibited similar stem cell properties, as reflected from the genes involved in pluripotency and differentiation (Table 4) expressing at similar levels, suggesting their origin from the hepatic stem cells.

**Table 4:** Expression of pluripotency, differentiation and housekeeping genes in CD90^+^NTSCs and CD90^+^CSCs.

Genes	Mean expression (RPKM)	
	CD90+NTSCs	CD90+CSCs
**Pluripotency and differentiation**		
Nanog	57	61
Oct3/4	2.6	1.6
Sox18	18	15
**Housekeeping**		
ACTB	1475	2385
GAPDH	128	179
HPRT1	3.4	2.1
PGK	28	24

No remarkable difference was observed between these two groups of CD90^+^ cells. Read counts were expressed in RPKM.

Housekeeping genes were expressed at comparable levels in CD90^+^CSCs and CD90^+^NTSCs, indicating that the expression changes of other genes could be reasonably compared between these two groups (Table 4).

Representative features of differentially expressed genes in CD90^+^CSCs and CD90^+^NTSCs were summarized (Table 5, up-regulated and Table 6, down-regulated). The up-regulated genes were associated with biological functions including drug transport (ABCC5), lipid metabolism (APOE, APOC1), angiogenesis (COL15A1, PLAU, PLVAP), cell proliferation (ESM-1, FGL1, GPC3, IGFBP5), transport (AMBP), acute inflammatory response (APOA2, ORM1), cytokine production (FABP4), cell cycle (PLK2), signal transduction (RAP2A), and activation of MAPK/ERK pathway (CXCR4). Down-regulated genes were involved in several biological processes including organ development (ADAMTS1, ALDH1A2), response to hypoxia (ANGPTL4, EDN1, SOCS3, VEGFA), nucleotide binding (ATP2A3, RAN, RPLP2), translation elongation activity (EEF1D), and chemotaxis (IL8, CXCL1).

**Table 5:** List of up-regulated genes in CD90^+^CSCs as compared with CD90^+^NTSCs.

GeneId	GeneSymbol	Gene Name	log_2_ ratio	*P* value	FDR	Function
NM_130786	A1BG	Alpha-1-B glycoprotein	4.645	0.026	0.032	Plasma glycoprotein
NM_001023587	ABCC5	ATP-binding cassette, sub-family C (CFTR/MRP), member 5	1.069	0.037	0.041	Drug efflux transporter
NM_001615	ACTG2	Actin, gamma 2, smooth muscle, enteric	6.495	0.001	0.002	Cell motility
NM_001633	AMBP	Alpha-1-microglobulin/bikunin precursor	1.531	0.000	0.000	Precursor of urinary trypsin inhibitor and lipocalin transport protein
NM_001643	APOA2	Apolipoprotein A2	2.434	0.000	0.000	Stabilize high density lipoprotein (HDL) structure and HDL metabolism
NM_000041	APOE	Apolipoprotein E	2.519	0.002	0.004	Lipoprotein catabolism, binding and internalization
NM_001645	APOC1	Apolipoprotein C1	1.601	0.000	0.000	Modulate lipoprotein interactions
NM_152547	BTNL9	Butyrophilin-like protein 9 precursor	1.693	0.000	0.000	Membrane-based protein with unknown function
NM_001855	COL15A1	Collagen, type XV, alpha 1	2.858	0.013	0.019	Structural protein
NM_001008540	CXCR4	Chemokine (C-X-C motif) receptor 4	2.663	0.000	0.000	Receptor specific for stromal cell-derived factor-1
NM_001135604	ESM1	Endothelial cell-specific molecule 1	2.590	0.018	0.024	Lung endothelial cell-leukocyte interactions and endothelium-dependent pathological disorders
NM_001442	FABP4	Fatty acid binding protein 4, adipocyte	2.183	0.000	0.000	Fatty acid uptake, transport, and metabolism
NM_004467	FGL1	Fibrinogen-like 1	2.832	0.000	0.000	Hepatocyte mitogenic activity, HCC development
NM_004484	GPC3	Glypican 3	2.686	0.026	0.032	Control of cell division and growth regulation
NR_002196	H19	H19, imprinted maternally expressed transcript (non-protein coding)	2.900	0.000	0.000	Tumor suppression
NM_000412	HRG	Histidine-rich glycoprotein	1.713	0.031	0.036	Blood coagulation
NM_000599	IGFBP5	Insulin-like growth factor binding protein 5	2.992	0.000	0.000	Prolong the half-life of the IGFs and regulate the growth promoting effects of IGFs
NM_002215	ITIH1	Inter-alpha (globulin) inhibitor H1	2.536	0.018	0.024	Hyaluronan synthesis, binding and transport and stimulation of phagocytotic cells
NM_002217	ITIH3	Inter-alpha (globulin) inhibitor H3	4.975	0.013	0.019	Extracellular matrix stabilization
NM_002291	LAMB1	Laminin, beta 1	1.633	0.037	0.041	Cell adhesion, differentiation and migration
NM_005947	MT1B	Metallothionein 1B	6.818	0.000	0.000	Bind heavy metals
NM_000607	ORM1	Orosomucoid 1	2.699	0.000	0.000	Unknown but suspected to be linked to immunosuppression
NM_001145031	PLAU	Plasminogen activator, urokinase	2.348	0.032	0.037	Degradation of the extracellular matrix
NM_006622	PLK2	Polo-like kinase 2	2.320	0.000	0.000	Regulation cell cycle progression, mitosis, cytokinesis, and DNA damage response
NM_031310	PLVAP	Plasmalemma vesicle associated protein	1.804	0.000	0.000	Formation of stomatal, microvascular permeability and fenestral diaphragms
NM_015869	PPARG	Peroxisome proliferator-activated receptor gamma	2.501	0.010	0.015	Regulation of adipocyte differentiation
NM_012212	PTGR1	Prostaglandin reductase 1	1.629	0.023	0.029	Inactivation of the chemotactic factor, leukotriene B4
NM_021033	RAP2A	RAP2A, member of RAS oncogene family	2.110	0.031	0.036	GTPase activity
NM_001085	SERPINA3	Serpin peptidase inhibitor, clade A (alpha-1 antiproteinase, antitrypsin), member 3	1.509	0.031	0.036	Plasma protease inhibitor
NM_012339	TSPAN15	Tetraspanin 15	4.210	0.000	0.000	Regulation of cell development, activation, growth and motility

**Table 6:** List of down-regulated genes in CD90^+^CSCs as compared with CD90^+^NTSCs.

GeneId	GeneSymbol	Gene Name	log_2_ ratio	*P* value	FDR	Function
NM_006988	ADAMTS1	ADAM metallopeptidase with thrombospondin type 1 motif, 1	−3.275	0.000	0.000	Kidney development
NM_170697	ALDH1A2	Aldehyde dehydrogenase 1 family, member A2	−6.559	0.000	0.000	Liver development
NM_001039667	ANGPTL4	Angiopoietin-like 4	−1.900	0.000	0.000	Hypoxia
NM_005173	ATP2A3	ATPase, Ca++ transporting, ubiquitous	−3.683	0.019	0.025	Nucleotide binding
NM_133468	BMPER	BMP binding endothelial regulator	−4.778	0.003	0.005	Inhibitor of bone morphogenetic protein (BMP) function
NM_002982	CCL2	Chemokine (C-C motif) ligand 2	−6.608	0.000	0.000	Moncyte chemotaxis
NM_033027	CSRNP1	Cysteine-serine-rich nuclear protein 1	−2.647	0.001	0.002	Transcription factor regulation
NM_001554	CYR61	Cysteine-rich, angiogenic inducer, 61	−2.900	0.000	0.000	Cell adhesion
NM_001511	CXCL1	Chemokine (C-X-C motif) ligand 1 (melanoma growth stimulating activity, alpha)	−3.078	0.037	0.041	Chemotaxis
NM_001955	EDN1	Endothelin 1	−3.670	0.000	0.000	Response to hypoxia
NM_001130055	EEF1D	Eukaryotic translation elongation factor 1 delta (guanine nucleotide exchange protein)	−1.236	0.000	0.000	Translation elognation activity
NM_005438	FOSL1	FOS-like antigen 1	−3.834	0.001	0.002	Transcription factor regulation
NM_000518	HBB	Hemoglobin, beta	−2.335	0.000	0.000	Oxygen transporter activity
NM_181054	HIF1A	Hypoxia inducible factor 1, alpha subunit (basic helix-loop-helix transcription factor)	−1.788	0.048	0.049	Response to hypoxia
NM_033439	IL33	Interleukin 33	−2.085	0.011	0.016	Cytokine acitivity
NM_000600	IL6	Interleukin 6 (interferon, beta 2)	−7.169	0.000	0.000	Cytokine acitivity
NM_000584	IL8	Interleukin 8	−3.981	0.037	0.041	Chemotaxis
NM_002391	MDK	Midkine (neurite growth-promoting factor 2)	−1.832	0.002	0.004	Nucleotide binding Function
NM_006325	RAN	RAN, member RAS oncogene family	−2.153	0.000	0.000	RNA binding Function
NM_001004	RPLP2	Ribosomal protein, large, P2	−1.714	0.000	0.000	RNA binding Function
NM_004704	RRP9	Ribosomal RNA processing 9, small subunit (SSU) processome component, homolog (yeast)	−1.711	0.048	0.049	Processing of pre-ribosomal RNA
NM_003955	SOCS3	Suppressor of cytokine signaling 3	−1.591	0.000	0.000	Response to hypoxia
NM_003254	TIMP1	TIMP metallopeptidase inhibitor 1	−4.580	0.000	0.000	Extracellular matrix de
NM_016639	TNFRSF12A	Tumor necrosis factor receptor superfamily, member 12A	−3.592	0.001	0.002	Apoptosis
NM_080682	VCAM1	Vascular cell adhesion molecule 1	−3.633	0.000	0.000	Cell adhesion
NM_001025370	VEGFA	Vascular endothelial growth factor A	−3.399	0.037	0.040	Response to hypoxia

### Validation of RNA-Seq data by qRT-PCR

**Figure 2:**
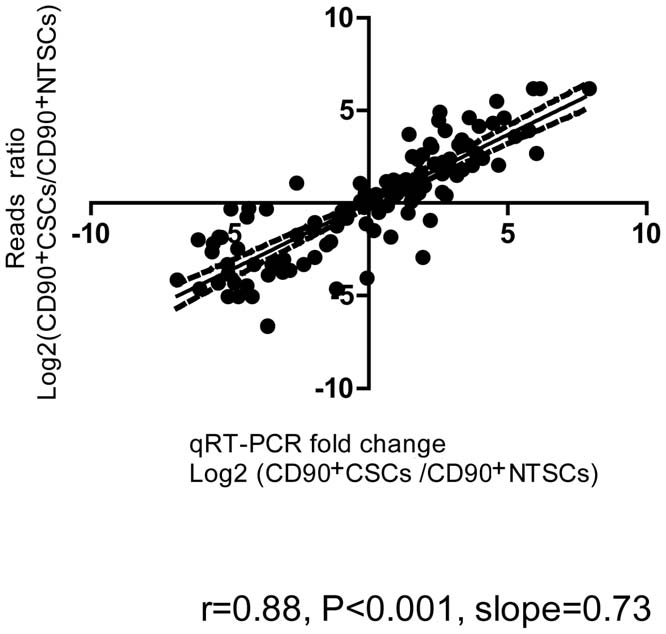
Correlation between qRT-PCR and RNA-Seq data. Correlation between qRT-PCR and RNA-Seq data of 47 selected genes: 28 up-regulated genes and 19 down-regulated genes in 3 pairs of amplified RNA samples. Spearman Rank Correlation coefficient = 0.88 (*P*<0.001) and slope = 0.73.

To verify the RNA-Seq data, the original six amplified RNA samples used for RNA-Seq were tested again by qRT-PCR on a panel of 47 differential expressed genes. Selected genes included 28 up-regulated genes and 19 down-regulated genes. Log_2_ fold change of genes of qRT-PCR was compared with that of RNA-Seq. These two gene expression analysis platforms demonstrated concordant results (Spearman Rank Correlation = 0.88, *P*<0.001; Figure 2). In addition, the slope of the regression line was 0.73, suggesting that RNA-Seq had a similar dynamic range of detection as that of qRT-PCR, and hence our RNA-Seq method could reliably measure gene expression differences, particularly for those lowly expressed genes in the CD90^+^CSCs and CD90^+^NTSCs.

### Confirmation of RNA-Seq data by qRT-PCR using samples from an independent patient cohort

**Figure 3:**
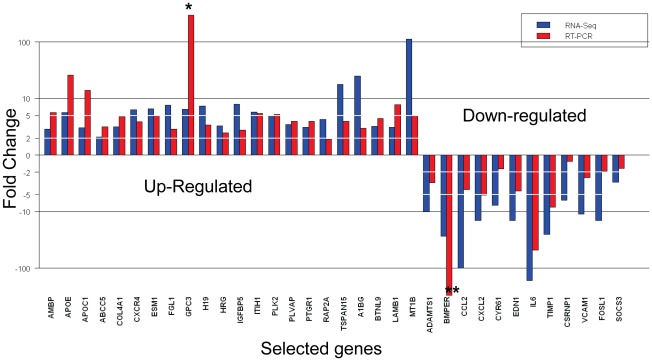
Prospective validation of RNA-Seq analysis using an independent cohort of 12 patients by qRT-PCR. Twenty-seven up-regulated genes and 15 down-regulated genes were selected for validation. The fold changes of selected genes measured by qRT-PCR were statistically significant (*P*<0.05). Gene expression difference was considered to be valid if the direction of change was the same (as estimated by RNA-Seq analysis). The percentage of concordance of qRT-PCR with the change of direction estimated by RNA-Seq analysis for the selected genes was 80%. *: The expression of GPC3 in CD90^+^NTSCs was not detected and its fold change could not be calculated. Further analysis by Fluidigm digital array confirmed the finding. **: The expression of BMPER in CD90^+^CSCs was not detected. Further analysis by Fludigim digital array confirmed the finding.

To eliminate potential bias as a result of pre-amplification and to further validate the RNA-Seq results, qRT-PCR of 27 up-regulated genes and 15 down-regulated genes was performed in 12 pairs of RNA samples prepared from CD90^+^CSCs and CD90^+^NTSCs derived from an independent batch of tumor and parallel non-tumor tissues, respectively. None of these samples underwent RNA amplification nor assayed for RNA-Seq analysis. A gene expression difference was considered to be valid if the trend of change of a gene measured by qRT-PCR agreed with that determined by the RNA-Seq analysis. Twenty-two out of 27 (81.5%) selected up-regulated genes were concordant with the trend estimated by RNA-Seq, whereas 12 out of 15 (80.0%) selected down-regulated genes were correlated with the down-regulated pattern estimated by RNA-Seq (Figure 3). This high concordant result suggested that RNA amplification did not introduce bias to the results of the gene expression profiling under study.

**Figure 4:**
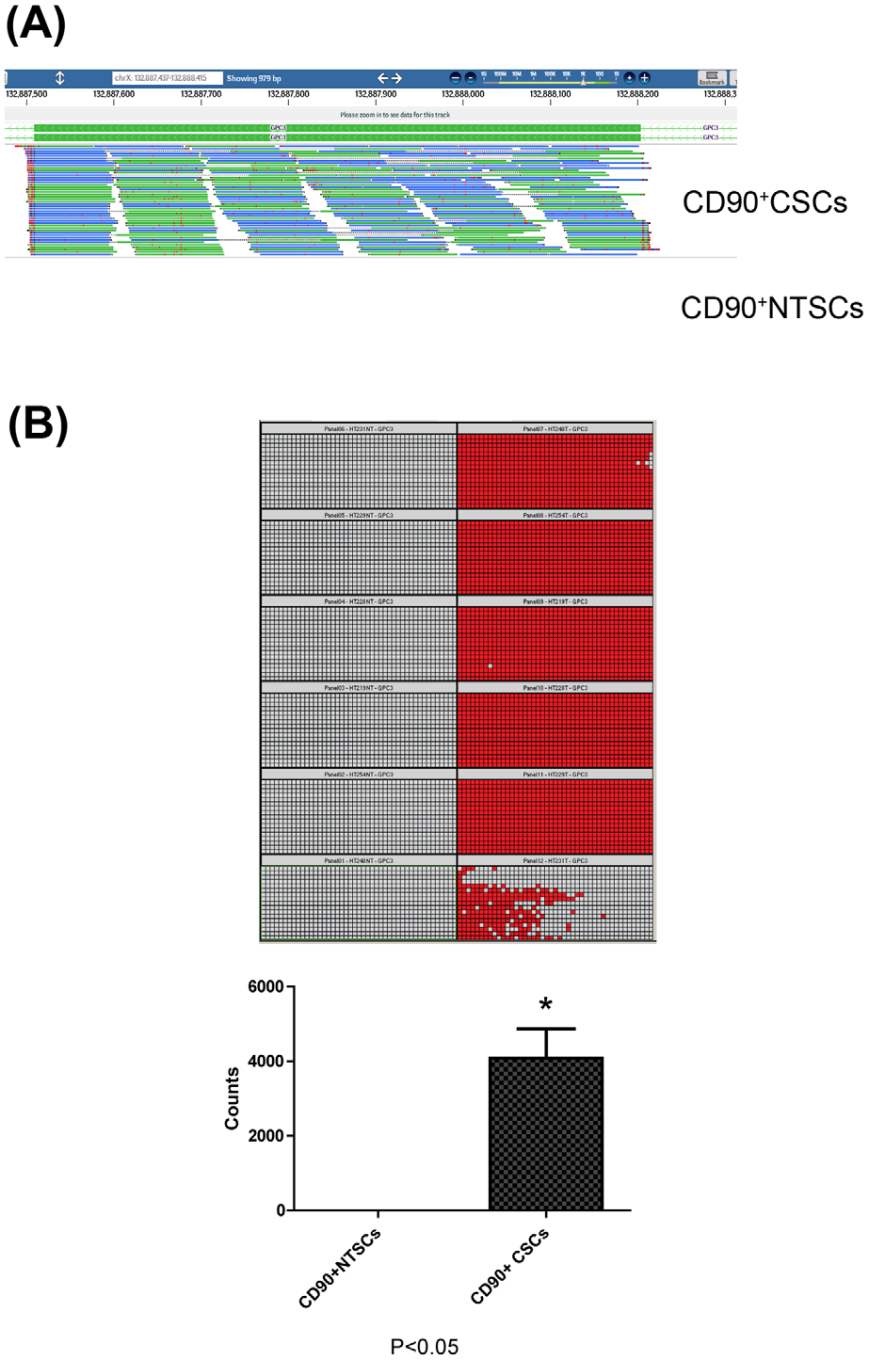
Read distribution along the GPC3 gene and quantitative measurement of mRNA GPC3 by Fluidigm digital array assay. (A) Alignment of RNA-Seq sequence reads to GPC3 gene. Significantly higher read counts were detected for CD90^+^CSCs when compared with those of CD90^+^NTSCs, indicating the specificity of GPC3 in liver CD90^+^CSCs. For illustration purpose, only one exon of the gene was shown. (B) Each digital array chip can run twelve samples. The six samples of the right hand side of the chip were CD90^+^CSCs, and of the left hand side were the corresponding CD90^+^NTSCs. Digital array partitioned a RNA sample premixed with RT-PCR reagents into individual 765 RT-PCR reactions. In each partition, the red color indicated positive expression of GPC3 at mRNA level, whereas grey indicated no expression. The GPC3 mRNA level was quantified by counting the positive signals by the software. The mRNA expression of GPC3 was predominantly expressed in CD90^+^CSCs as compared with CD90^+^NTSCs (*P*<0.05).

We found that APOE, ESM-1, H19, ITIH1, PLVAP, PLK2, and LAMB1 were highly expressed in CD90^+^CSCs compared to CD90^+^NTSCs (*P*<0.01). The expression of GPC3 was unambiguously confined to CD90^+^CSCs and not detected in most of the CD90^+^NTSCs (Figure 4A). The absence of GPC3 in CD90^+^NTSCs was further demonstrated by Fluidigm digital array in human HCC tissues (*P*<0.05; Figure 4B).

### Functional annotation of differentially expressed genes by gene ontology analysis

To more systematically examine the enriched genes related to the liver CSCs, we used the gene ontology (GO) enrichment analysis to functionally annotate and predict the biological roles of these differentially expressed genes. We performed the GOTM ((http://bioinfo.vanderbilt.edu/gotm/goanalysis_page_one.php) and ProfComp (http://webclu.bio.wzw.tum.de/profcom/start.php) analysis for clustering the genes with related biological functions (Table 7). Up-regulated genes in liver CSCs were associated with biological processes such as response to external stimulus (CXCR4, APOE, LAMB1), response to chemical stimulus (ABCC5, CRP, APOH, KNG1), inflammatory response (CRP, TF, C4BPB), homeostasis (SERPIND1, APOE, HRG), cholesterol transport and phospholipid efflux (APOE, APOC1, APOA1). Besides, the 43 up-regulated genes, present in extracellular regions and space, including COL4A1, PLVAP, IGFBP5, GPC3, ITIH1 and ESM-1, were involved in remodeling of extracellular matrix. Being a critical component of tumor environment, extracellular matrix was important for the production of secretory proteins which affected various biological activities, such as metastasis. On the other hand, down-regulated genes in CD90^+^CSCs were those over-expressed in CD90^+^NTSCs. These over-expressed genes were related to biological processes, such as translational elongation (RPL39, EEF1B2, FAU), cell motion (IL-8, EDN1, CCL2, IL6), anti-apoptosis (SOCS3, IL-6, HSPB1), negative regulation of cellular process (TIMP1, BIRC3, VEGFA), angiogenesis (Jun, VEGFA, ANGPTL4), and cell proliferation.

**Table 7:** Enrichment of genes involved in biological process in CD90^+^CSCs.

Gene Ontology	Number of genes	Adjusted *P* value
**Up-regulated genes**		
Response to external stimulus	32	3.05E-10
Response to wounding	26	3.05E-10
Acute phase response	9	5.46E-10
Response to chemical stimulus	10	5.46E-10
Inflammatory response	19	1.69E-08
Phospholipid efflux	6	2.95E-09
Cholesterol transport	6	7.88E-08
Regulation of small RNA production	18	7.88E-08
Homeostatsis	12	7.88E-08
**Down-regulated genes**		
Translational elongation	30	1.07E-20
Cell motion	44	2.32E-09
Cell localization	44	2.32E-09
Anti-apoptosis	26	3.84E-09
Negative regulation of cellular process	75	8.12E-09
Angiogenesis	22	8.94E-09

Only categories with three or more candidate genes are shown.

### Expression of GPC3 and quantity of CD45^−^CD90^+^ cells in HCC tumor tissues

**Figure 5:**
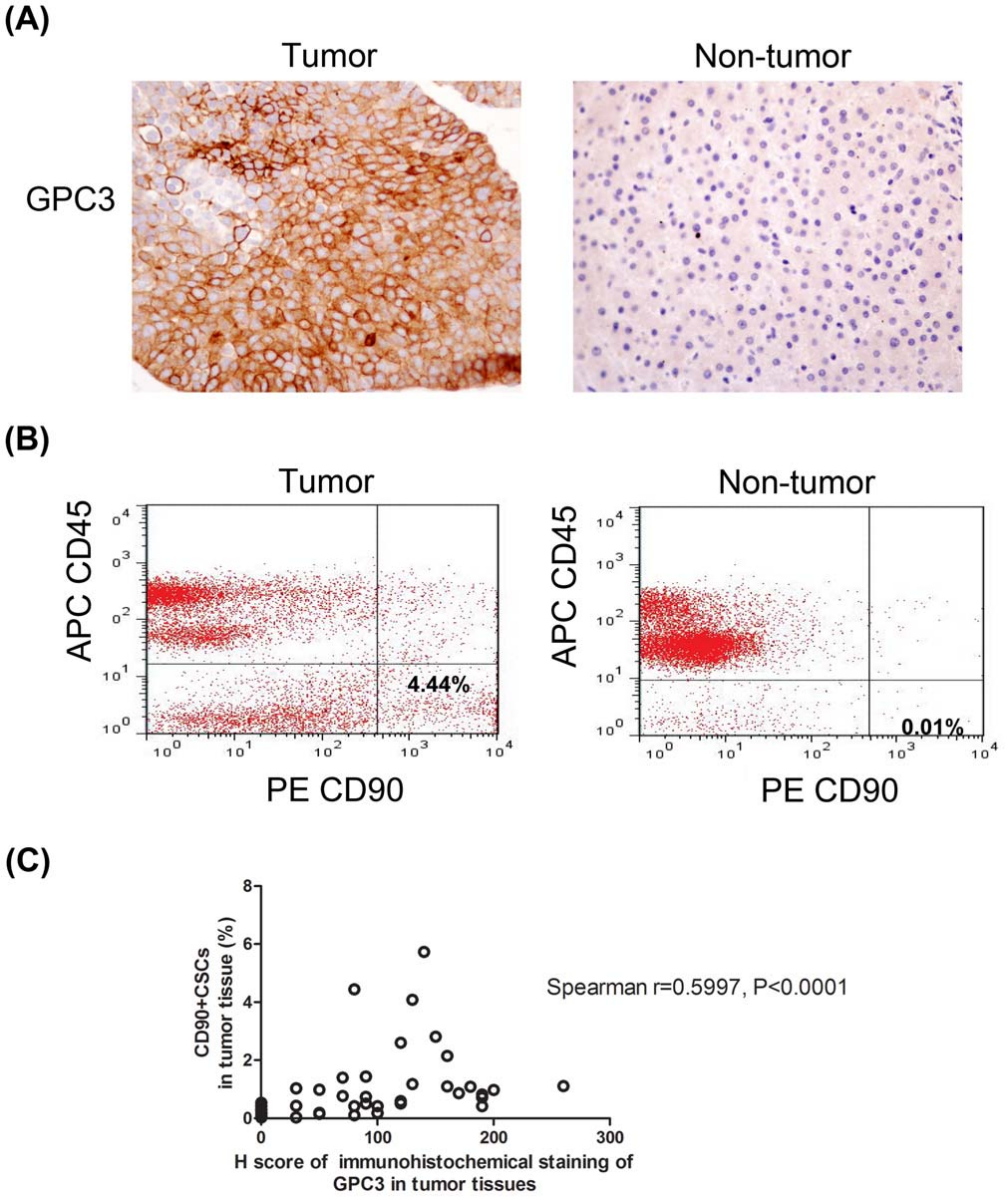
GPC3 expression and quantification of CD90^+^ cells in human liver tumor tissues. (A) Immunohistochemistry detected strong signals of GPC3 in liver tumor tissue, but negative staining for GPC3 was detected in the adjacent non-tumorous tissue (magnification×200). (B) Flow cytometry detected more CD45^−^CD90^+^ cells in tumor tissues (median, 0.645%; range, 0.06–4.59% of the gated cells) than that in adjacent non-tumorous tissues (median, 0.175%; range, 0.00–1.14%). (C) The number of CD45^−^CD90^+^cells was positively correlated with GPC3 expression level in the tumor tissues (Spearman correlation coefficient = 0.5997, *P*<0.0001).

Among the genes identified by RNA-Seq, GPC3 was selected for further investigation because it is uniquely expressed in liver CD90^+^CSCs but absent in CD90^+^NTSCs. Immunohistochemistry revealed strong GPC3 expression in 34 out of forty-two HCC tumor tissues (80.9%), but no expression was detected in non-tumorous tissues (Figure 5A). This result was in concordance with the previous findings that GPC3 was differentially expressed in HCC [31].

Our results also showed the presence of liver CD45^−^ CD90^+^ cancer stem cells in all forty-two HCC tumor tissues (0.06–4.59% in gated cells) in flow cytometry (Figure 5B), indicating a high specificity of CD90^+^CSC cells in HCC. Interestingly, a significant positive correlation between GPC3 expression and CD45^−^CD90^+^ cancer stem cells was found in the HCC tumor tissues (Spearman r = 0.5997, *P*<0.0001; Figure 5C).

### Expression of CD90^+^GPC3^+^ cells in HCC cell lines and human HCC tissues

**Figure 6:**
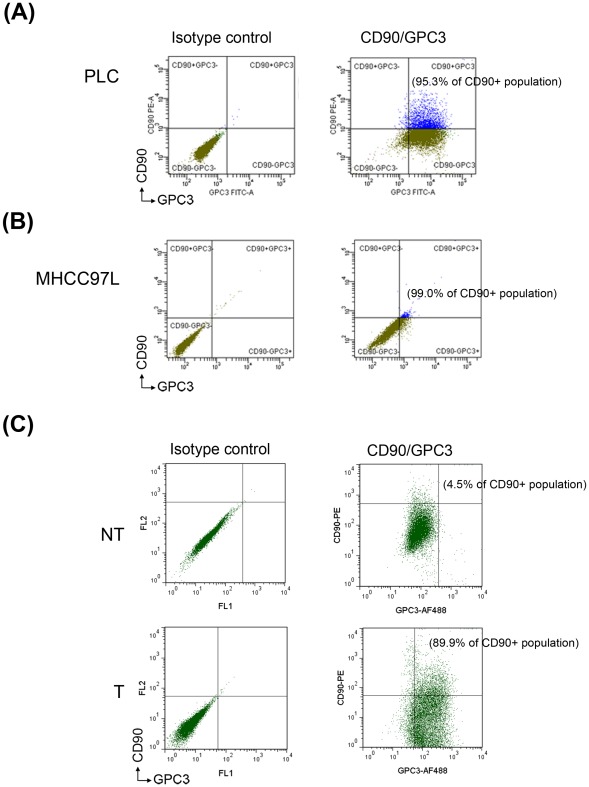
High prevalence of CD90^+^GPC3^+^ cells in CD90^+^CSCs derived from human HCC cell lines and liver tumors. A significant increase in the number of CD90^+^GPC3^+^ cells were detected within CD90^+^ cell population of PLC and MHCC97L cells. (A) In PLC cells, 95.3% of CD90^+^ cells co-expressed GPC3. (B) In MHCC97L cells, 99.0% of CD90^+^ cells co-expressed GPC3. (C) Analysis of a representative pair of human liver tissues indicated that only 4.5% of CD90^+^ population expressed GPC3 in non-tumorous tissues, while 89.9% of CD90^+^ cells expressed GPC3 in the matched tumorous tissues (median, 86.4%; range, 54.2–91.0%; n = 5). These results demonstrated that GPC3 is distinctly expressed in liver CD90^+^CSCs.

To validate the specificity and abundance of GPC3 in CD90^+^CSCs, two-color flow cytometry was used to measure the expression of total GPC3 (both cell membrane and cytoplasm) [31] in CD90^+^CSCs derived from two HCC cell lines. GPC3 was distinctly expressed in CD90^+^ cells derived from PLC cell line (95.3%, Figure 6A) and MHCC97L cell line (99.0%, Figure 6B). This result indicated predominant expression of GPC3 in liver CSCs. Further study on human HCC tissues also demonstrated that GPC3 was highly expressed in liver CD90^+^CSCs (median, 86.4%; range, 54.2–91.0%; n = 5; Figure 6C).

Owing to the high expression of GPC3 in liver cancer stem cells, additional functional studies were performed to investigate if GPC3 has pivotal roles in regulating the growth of liver cancer stem cells.

### GPC3 knockdown by siRNA

**Figure 7:**
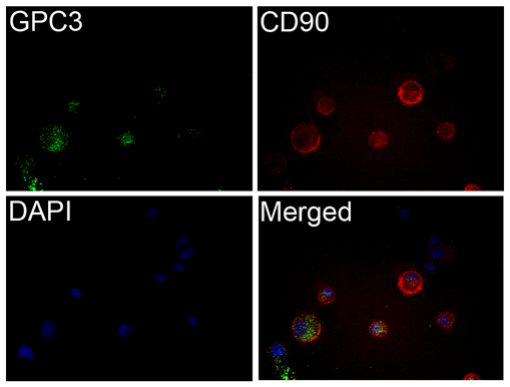
Double immunofluorescence staining of CD90 and GPC3 in sorted PLC CD90^+^GPC3^+^ cells. The sorted cells were stained with fluorescein-conjugated anti-CD90 and anti-GPC3 antibodies. Nuclei were counterstained by DAPI. The merge image showed the expression of CD90 and GPC3 in both cytoplasm and cell membrane.

**Figure 8:**
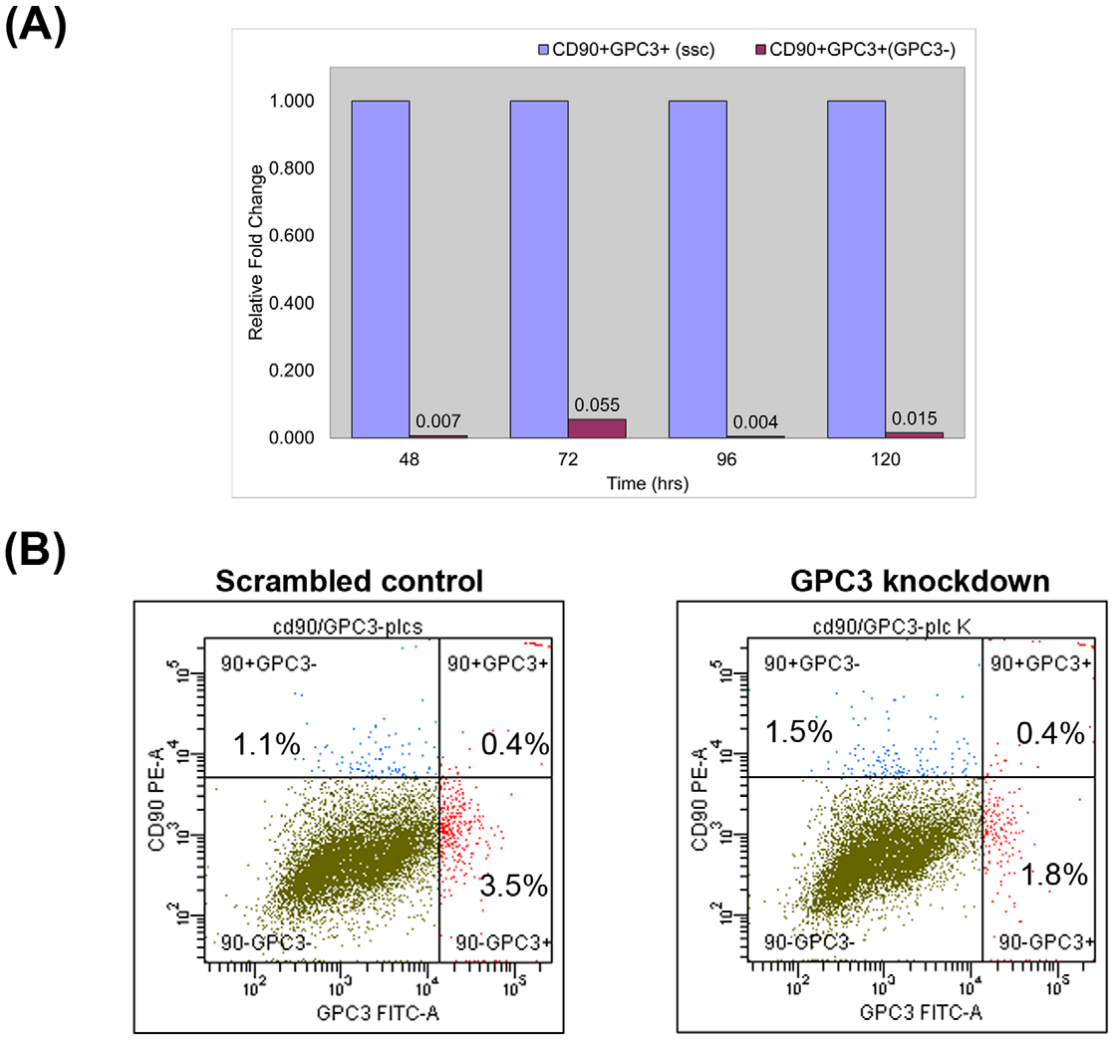
Effective knockdown of GPC3 in PLC CD90^+^GPC3^+^ cells. The sorted PLC CD90^+^GPC3^+^ cells were transfected with either 20 nM specific GPC3 siRNA or a scrambled siRNA control and incubated for 24 hours. (A) GPC3 knockdown in the target cells reduced the gene expression by 90% as measured by qRT-PCR. (B) By flow cytometry, the number of GPC3-expressing cells was decreased by 43% upon GPC3 knockdown when compared to the scrambled control (decreased from 3.9% to 2.2%).

PLC CD90^+^GPC3^+^ CSCs were sorted using FACS with the conjugated antibodies of FITC-GPC3 and PE-CD90 (Figure 7).We transfected PLC CD90^+^GPC3^+^ cells with either GPC3-specific siRNA or scrambled control. The efficacy of knockdown was determined by qRT-PCR at various time points. More than 90% inhibition of mRNA expression of GPC3 was achieved (Figure 8A). Consistently, by flow cytometry, the percentage of GPC3 expressing cells was reduced by more than 43% after GPC3 knockdown when compared to the negative control (Figure 8B). There was no remarkable change in the percentage of CD90^+^CSCs in PLC cells upon GPC3 transfection, implicating that GPC3 suppression has no effect on the liver CD90^+^CSCs.

### Effect of GPC3 on cell proliferation and colony formation of liver CD90^+^CSCs cells

**Figure 9:**
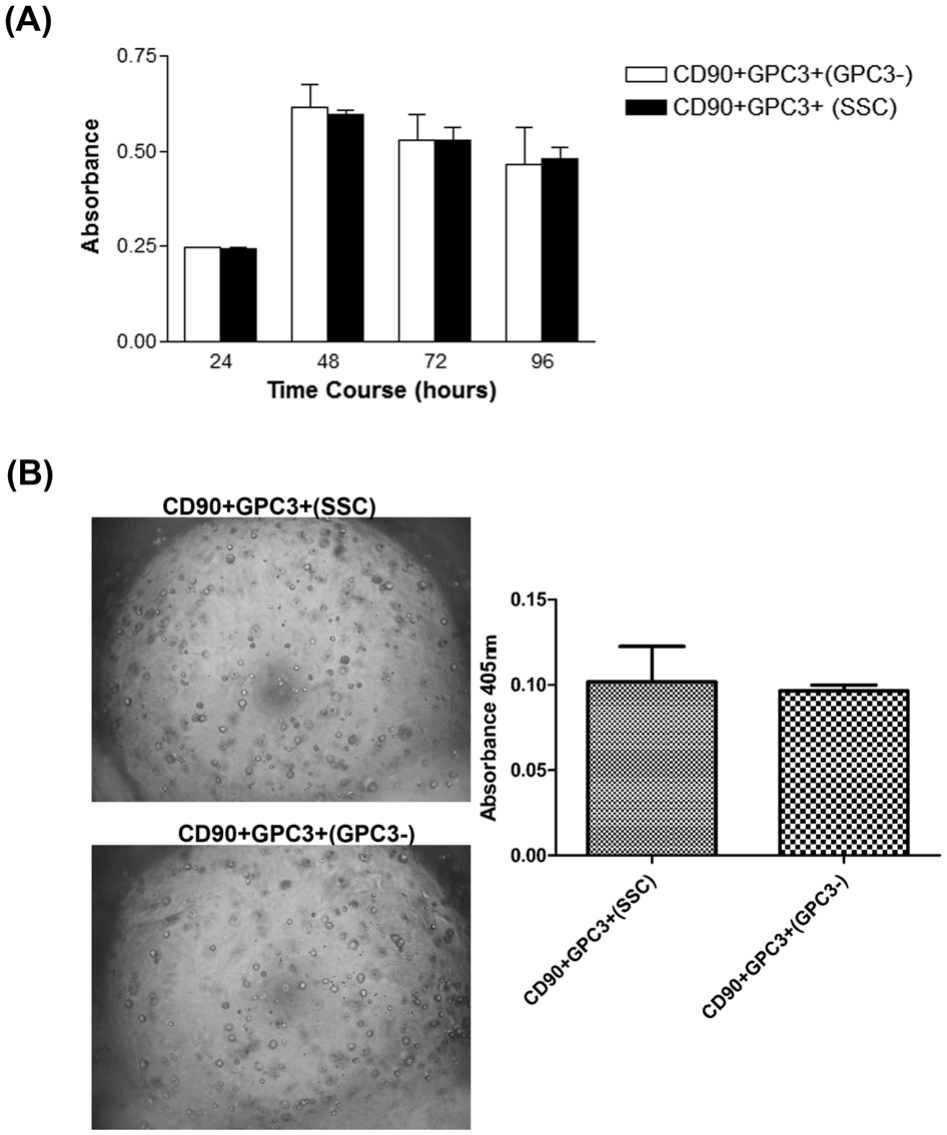
Effect of GPC3 on cell proliferation and clonogenic capacity of liver CD90^+^GPC3^+^CSCs. (A) Cell proliferation was assessed after GPC3 knockdown in PLC CD90^+^GPC3^+^ cancer stem cells. No significant effect of GPC3 on liver cancer stem cell proliferation was found. (B) Knockdown of GPC3 in PLC CD90^+^GPC3^+^ cancer stem cells by siRNA did not affect their colony formation ability, indicating that GPC3 had no impact on clonogenicity of the liver cancer stem cells.

When the proliferation rate was compared between PLC CD90^+^GPC3^+(GPC3−)^ and CD90^+^GPC3^+^^(SSC)^ over a 4-day period, we noted no difference in cell proliferation between these two groups of cells. This suggested that the GPC3 does not play a role in regulating cell proliferation of the liver CD90^+^CSCs (Figure 9A).

Clonogenicity is one of the fundamental properties of cancer stem cell, which was measured in PLC CD90^+^GPC3^+(GPC3−)^ and PLC CD90^+^GPC3^+^^(SSC)^ cells. As shown in Figure 9B, both types of cells had similar clonogenic capacity.

Taking the results of these functional studies together, we suggest that GPC3 is not involved in regulating the studied properties of liver cancer stem cells.

## Discussion

The current strategy of the new anti-cancer therapies focuses on complete eradication of CSCs by targeting at Wnt/ß-catenin, Hedgehog and Notch pathways which play critical roles in the self-renewal process of CSCs. Because these developmental signaling cascades also interact with other pathways in normal biological functions, concerns are raised that normal stem cells will be unavoidably damaged due to non-specificity of current anti-cancer therapies [32]. Therefore, specific targeting of CSCs while preserving their normal counterparts should be the appropriate approach, but the success relies on identification of specific target genes in CSCs. In this study, the gene expression differences between CD90^+^CSCs from tumor tissue and CD90^+^NTSCs from non-tumorous counterparts were identified by RNA-Seq, and the up-regulated genes in CD90^+^CSCs were associated with the biological processes of liver inflammation, chemoresistance and lipid metabolism.

Emerging evidence show that chronic inflammatory disorders predispose to cancer development, but the underlying mechanisms are not fully understood. HCC is a typical inflammation-related cancer that develops slowly on a background of chronic liver inflammation, mainly triggered by the hepatitis virus, such as hepatitis B and C. The host immune system is activated upon inflammation and creates inflammatory microenvironment composing of leukocyte infiltrates, activated resident macrophages and the consistent generation of cytokines within the diseased liver [33]. In this study, a higher expression of genes in CD90^+^NTSCs that facilitated the activation of resident macrophages, recruitment of inflammatory cells and release of cytokines for attempting to eliminate viral-infected liver cells as a protective mechanism were found. However, the host adaptive immune response to viral hepatitis is not sufficient to get rid of infection completely, leading to persistent immune-mediated liver injury, which becomes an important procarcinogenic factor.

Additionally, these immune responses may even facilitate growth, progression and metastasis of tumor cells [33]. Further, the up-regulated genes in CD90^+^CSCs were associated with biological processes of wound healing, inflammation, and response to external stimuli that might aggravate chronic liver inflammation. As a result, an inflammatory microenvironment was established that accelerates tumor growth and progression. Interestingly, the gene expression of IL6 and CCL2, which regulate cell motility, was low or even absent in CD90^+^CSCs, but were highly expressed in CD90^+^NTSCs, suggesting that liver CSCs might attempt to escape immune attacks by monocyte infiltrations. It has been shown that excessive monocyte infiltrations were associated with tumor regression [34]. It appears that the overall effects of liver inflammation favor survival and progression of CD90^+^CSCs. Hence, it is reasonable to believe that anti-inflammatory therapies would induce adverse effects on the activities of CD90^+^CSCs. Besides, the over-expressed genes in CD90^+^CSCs were associated with responses to chemical stimulus. One of the genes involved in this category, ABCC5, is a membrane-bound protein drug transporter and is up-regulated in HCC cells [35]. The over-expression of ABCC5 gene results in elevated production of drug transporter proteins, which protect the liver CSCs from chemotherapeutic drugs by pumping drugs out of the cells.

In our study, genes that are associated with the lipid metabolism were more prominent in CD90^+^CSCs as compared with CD90^+^NTSCs. A recent finding has demonstrated that genes involved in the lipid metabolism are crucial in cell transformation and over-expressed in tumor tissues [36]. Diabetes and non-alcoholic fatty liver disease may significantly increase the risk of developing HCC when other risk factors, such as hepatitis B virus, are present [37]. It is speculated that metabolic diseases, such as diabetes mellitus, and cancer may share common disrupted metabolic pathways [36]. In our study, APOE and APOC1 were highly expressed in CD90^+^CSCs as compared with CD90^+^NTSCs, but their functional attributes in liver CSCs remained to be clarified. Nevertheless, APOE is known to be involved in signal transductions that are important in tumor cell survival, proliferation and migration [38]. Elevation of APOC1 expression was associated with tumor cell survival in pancreatic cancer [39]. Because cholesterol is an essential component of cell membranes, rapid growth and cell division of tumor cells depend on cholesterol availability. In addition, cholesterol trafficking influences the generation of essential signaling intermediates, such as arachidonic acid, which are crucial in regulating cellular activities. Therefore, the CD90^+^CSCs with active genes in lipid transport are believed to facilitate its proliferation, differentiation, growth and progression.

The level of endothelial cell specific molecule-1 (ESM-1) mRNA was highly expressed in CSCs, which was aligned with its function in inflammatory responses and tumor progression [40]. In addition, ESM-1 is associated with angiogenesis [41]. In this study, concomitant up-regulated expression of ESM-1 mRNA with PLVAP mRNA in CSCs was found. Evidence has demonstrated that PLVAP expression is regulated by VEGF signaling and interacts with tumor angiogenesis [42]. Taken these together, simultaneous elevation of ESM-1 and PLVAP in CSCs suggested that CSCs is crucial for HCC development through promoting angiogenesis.

Among the genes identified, we found that the mRNA level of GPC3 is nearly absent in the CD90^+^NTSCs, but is highly expressed in CD90^+^CSCs. Recent studies have shown over-expression of GPC3 in HCC [43], [44]. GPC3 is highly expressed in fetal liver, but gradually the expression is decreased towards birth [45]. Interestingly, GPC3 expression resumes in the HCC tissue during hepatic carcinogenesis [45]. In our present study we also found a positive correlation between GPC3 expression and the number of CD90^+^CSCs present in the liver tumor tissues, suggesting that GPC3 expression could indicate the amount of liver cancer stem cells in tumors. Owing to the small number of cases and short follow-up period, correlation analysis of survival or other clinico-pathological features with the number of CD90^+^CSCs or GPC3 could not be accurately evaluated. Nevertheless, a previous study using the same protocol of GPC3 immunohistochemical staining has shown that GPC3-positive HCC patients (>10% of GPC-positive cells in immunohistochemical staining) have a lower 5-year survival rate than GPC-negative HCC patients (<10% of GPC-positive cells in immunohistochemical staining [46]. Taken our results together with their findings, GPC3 expression in liver tumor tissues may reflect the abundance of liver cancer stem cells, which may have a prognostic value for HCC patients. Additionally, it was shown that GPC3 was highly expressed in CD90^+^CSCs in liver cancer cell lines and human tumor tissues, implicating that GPC3 is a specific marker for liver CD90^+^CSCs. Some studies demonstrated that GPC3 plays an important role in the cell growth and differentiation by interacting with heparin-binding growth factors, such as IGF and Wnts [47], [48]. Besides, GPC3 has been shown to induce ERK1/2 and AKT phosphorylation that eventually contributes to anti-apoptosis, invasion, and survival of tumor cells [49]. On the other hand, some other studies reported that endogenous GPC3 inhibited the cell proliferation of liver cancer cells [50], [51]. Another recent study demonstrated that GPC3 acts as a negative regulator of cell proliferation, but this mechanism may be defective in HCC and thereby cancer cells are unresponsive to over-expressed GPC3 signal [52]. All these studies suggested that the role of GPC3 in HCC is elusive. In this study, we showed that GPC3 did not have a regulatory role in cell proliferation of liver cancer stem cells. The exact role of GPC3 on cancer stem cells therefore needs further investigation.

Regardless of its ambiguous functional roles on HCC, GPC3 is a potential target gene for liver cancer therapy because it is highly expressed in HCC but is absent in normal liver tissues. Making use of this distinct feature of GPC3 in HCC, an anti-GPC3 antibody therapy or antibody-drug conjugate (ADC) could be used as a biological missile targeting the GPC3-expressing cells, including liver cancer cells [53] and CD90^+^CSCs. The binding of the target cells by ADC target therapy would release the linked cytotoxic drug once the conjugate is internalized into the cells and eventually eradicate both cancer cells and CSCs. In a recent study, the GPC3-antibody approach aroused antibody-dependent cellular cytotoxicity, which caused liver tumor growth retardation in an animal model [54]. The phase I clinical trial using GPC3 for liver cancer is ongoing [55]. Elimination of liver cancer stem cells could be potentially achieved by this molecular targeted therapy.

In summary, the present study has identified a set of genes differentially expressed by liver CSCs that are enriched in several biological processes including inflammation, drug resistance and lipid metabolism using RNA-Seq. Additionally, the genes that are distinctly expressed in liver CD90^+^CSCs but not in CD90^+^NTSCs, such as GPC3, could be promising candidates for immunotherapy, which could eliminate liver cancer stem cells and adult cancer cells without inducing damage to normal liver cells. The present study provides clues for the development of new drugs targeting against liver CSCs and may lead to an improvement in the outcomes of HCC patients.

## Supporting Information

Figure S1 **CD90^+^ cells sorting.** CD90^+^ cells were sorted from tumor and adjacent non-tumorous human liver tissues using a BD FACSAria II Cell Sorter. The purity of CD90^+^ was about 86.6%.
(TIF)

Table S1 **Primers used in qRT-PCR for the validations of RNA-sequencing data.**
(DOC)
