# Supplementary figures and images for: Gene Expression Profiling of Liver Cancer Stem Cells by RNA-Sequencing
Source: PLoS One. 2012 May 14;7(5):e37159. doi: 10.1371/journal.pone.0037159 (PMC3351419; doi:10.1371/journal.pone.0037159)

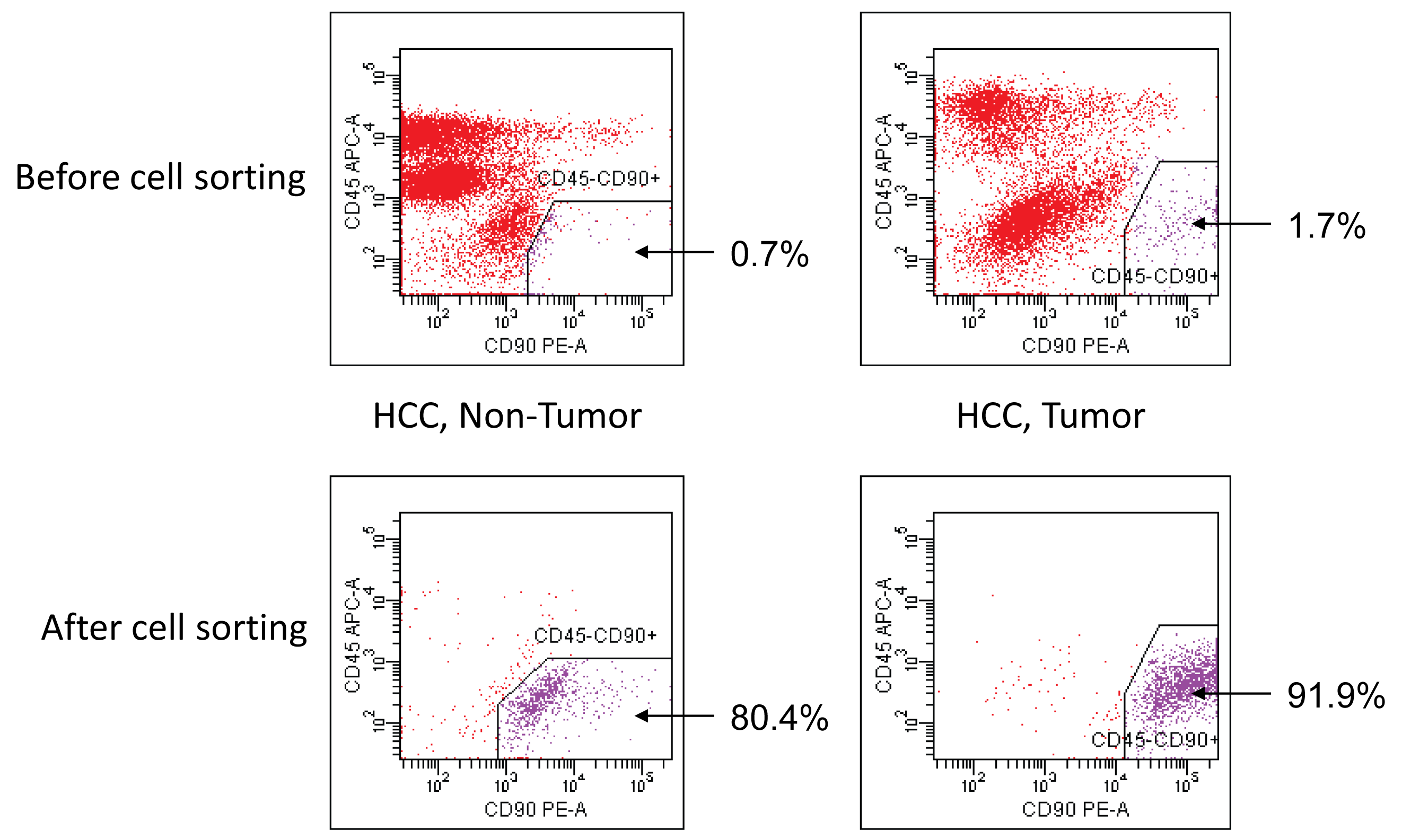

Supplement: Figure S1 — CD90+ cells sorting. CD90+ cells were sorted from tumor and adjacent non-tumorous human liver tissues using a BD FACSAria II Cell Sorter. The purity of CD90+ was about 86.6%. (TIF) [file pone.0037159.s001.tif]
