# Supplementary material for: Gene Expression Profiling of Liver Cancer Stem Cells by RNA-Sequencing
Source: PLoS One. 2012 May 14;7(5):e37159. doi: 10.1371/journal.pone.0037159 (PMC3351419; doi:10.1371/journal.pone.0037159)
Supplement: Table S1 — Primers used in qRT-PCR for the validations of RNA-sequencing data. (DOC) [file pone.0037159.s002.doc]

**Table S1**. Primers used in qRT-PCR for the validations of RNA-sequencing data.

| **Gene** | **Gene Name** | **Forward primer** | **Reverse primer** |
| --- | --- | --- | --- |
| A1BG | Alpha-1-B glycoprotein | CTCTTGCTGTGGGGTGTCACCTGG | GCTCCTGGGCCACCCCATTCTT |
| ABCC5 | ATP-binding cassette, sub-family C (CFTR/MRP), member 51 | TGGCTGGCTTCAGTGGACCAAATTTT | GCCTGTTCCCACACACCTCGG |
| ADAMTS1 | ADAM metallopeptidase with thrombospondin type 1 motif, 1 | TGGGGGTTGGGAAATGAAAAGTAGGA | AGCAGCCATAAACAGTGCTGGTCA |
| AMBP | Alpha-1-microglobulin/bikunin precursor | CTGCCAGCTGGGCTACTCGG | GGGAGATTGCAGGCCGCCAC |
| ANKS1A | Ankyrin repeat and sterile alpha motif domain containing 1A | AGTCTCAGGGTGACGTGGAGAAAG | GGTCAGATGATCGCCCGCTGG |
| APOC1 | Apolipoprotein C-I | GACAGGAATCTTGCCCATTCC | TTTCCCCACTCAGAATGTAGCA |
| APOE | Apolipoprotein E | TGGCACTGGGTCGCTTTTGGG | TCATGGTCTCGTCCATCAGCGC |
| BIRC3 | Baculoviral IAP repeat-containing 3 | GCCTCTGGGCAGCAGGTTTACAA | CATGCACAAAACTACCTCCCGAGATTA |
| BMPER | BMP binding endothelial regulator | TTGCACTGAAAAGCCCGGCGT | CGAGAAGGAGCGTGTCCGGC |
| BTNL9 | Butyrophilin-like 9 | GGTGAGGAGCACACCAAGGAGTGA | CCGTGAGAAGCTTCATGGTGCTCT |
| C4BPB | Complement component 4 binding protein, beta | ACCAGGTGTCTGAGCTGGGTG | GGAGGAAGCTCTGGACAGTGCTCT |
| COL4A1 | Collagen, type IV, alpha 1 | TCTTCCTTTGAAAGACTGTGCTGTCC | ACCTGGGTTTGGTTTTGGCAACA |
| CRY61 | Cysteine-rich, angiogenic inducer, 61 | GGCCAGGATTGGAGGTAGGT | GTGAGAAGGTCTAAGAAAGGGTG |
| CSRNP1 | Cysteine-serine-rich nuclear protein 1 | AGCTTCACATCAGGCGTCCTGGA | CGTCCATGCTGGGTGGCACT |
| CTGF | Connective tissue growth factor | CTCGCGGCTTACCGACTGGAAGAC | CCACAGGTCTTGGAACAGGCGC |
| CXCL2 | Chemokine (C-X-C motif) ligand 2 | GGGGTTCGCCGTTCTCGGAG | ATGGGGCTCAGCAGGCGGTT |
| CXCR4 | Chemokine (C-X-C motif) receptor 4 | TTCCCTCTAGTGGGCGGGGC | TCCCCATCTTTTCCCATAGTGACTTCA |
| DUSP10 | Dual specificity phosphatase 10 | CCCGTCCGACCTCAGGATCTCAA | ACAACGGTGGTGGCGATGACA |
| EDN1 | Endothelin 1 | GCCAAGGAGCTCCAGAAACAGCA | GGAGCAGCGCTTGGACCGG |
| ESM1 | Endothelial cell-specific molecule 1 | GCTACCGCACAGTCTCAGGCA | GCAATCCATCCCGAAGGTGCCG |
| FGL1 | Fibrinogen-like 1 | AGGGGGAACATGGCAAAGGTGT | CCGCATCTGCTCCTGGGCAC |
| FMNL3 | Formin-like 3 | ACATTGGGTGGGTGCGGGAAT | GCACCATCGTCACCACTTTCCAGAC |
| FOSL1 | FOS-like antigen 1 | GCAGGCAGCCCAGCAGAAGT | ACTCCTGGCCGGGGTTGTGG |
| GSTA1 | Glutathione S-transferase alpha 1 | CGGTGACAGCGTTTAACAAAGCTTAGA | GGACTCCATTCTGCCCCGTGC |
| H19 | Imprinted maternally expressed transcript (non-protein coding) | TCGTGCAGACAGGGCGACATC | CCAGCTGCCACGTCCTGTAACC |
| HIGD1B | HIG1 hypoxia inducible domain family, member 1B | CCTCTCTCTAGGACGGGGCTGC | TCGTCGTCAGGTGGTACCCACC |
| HRG | Histidine-rich glycoprotein | CCCAGGTAAAGGACCCCGTCCC | TGTTTGTGGTGCGGCAATGGG |
| IER3 | Immediate early response 3 | TCTACCCTCGAGTGGTCCGGC | CGCCGGCACACCCTCTTCAG |
| IGFBP5 | Insulin-like growth factor binding protein 5 | CGGGAGAGGTGGGCGCTAGAA | GGAGACAGATCCGGGAGAGGTCC |
| IL-6 | Interleukin 6 | CTCCACAAGCGCCTTCGGTCC | TCAGGGCTGAGATGCCGTCGA |
| ITAG6 | Integrin, alpha 6 | TCGCTGGGATCTTGATGCTTGC | TGAGCATGGATCTCAGCCTTGTGA |
| ITIH1 | Inter-alpha (globulin) inhibitor H1 | GCCTTAGAGCATGGACGGTGCC | ACAGCCTGTCGCTTCTCGCTG |
| LAMB1 | Laminin, beta 1 | GCAAGCTGCAACTGCTCAAAGATTT | ACGGACTTCTCCTTCCAGTCTTGC |
| MAST4 | Microtubule associated serine/threonine kinase family member 4 | GGAGCGGCAGTGCCAGTGAG | CTGGCCCAAAGTGCCTCCCG |
| MGP | Matrix Gla protein | TGCGAACGCTACGCCATGGT | ACAAAATCAGGTGCCAGCCTCCA |
| MT1B | Metallothionein 1B | TCCTGCAAGAAGTGCTGCTGCTC | TGTAGCAAACCGGTCAGGGTAGTT |
| PLK2 | Polo-like kinase 2 | CCCAGCAACAGATGCTCCTGAGC | GGCAGATCTCCACCATCCATGAGG |
| PLVAP | Plasmalemma vesicle associated protein | GCCGAGGGCCTATACAGTCAGCT | GGTCGCGGCGAGCATTCAGC |
| PTGR1 | Prostaglandin reductase 1 | TGGCCCCACCCTCTCTTGGG | CCTGAAGCTCAGGAGCCCGA |
| PTGS1 | Prostaglandin-endoperoxide synthase 1 | GCCAGATTGCTGGCCGGATCG | GCTCCTGGAAGGAGGTGTAGGGT |
| RAP2A | RAP2A, member of RAS oncogene family | GGCAGATGAACTATGCTGCTCAGCC | GGCAAATCCCATCCAGGACAGCC |
| RBP1 | Retinol binding protein 1, cellular | GGCCTTGCGCAAAATCGCCA | CCGTCCCAGCTCACTGTTGTCATG |
| RBP7 | Retinol binding protein 7, cellular | CCGGGTTTGTCCCGCGATCC | TCTGTGGCTTCAGCAACTTGGCTA |
| RCAN1 | Regulator of calcineurin 1 | GCGGCGACTGGAGCTTCATTG | ACTCAAATTTGGCCCGGCACAG |
| RND1 | Rho family GTPase 1 | AAGCCTAGCCCACTGCCCCA | CGTGCCTCTGCACCCCAAGG |
| RPL23AP32 | Ribosomal protein L23a pseudogene 32 | TCCAGTCCCCTTCCTTCGGTGTT | TCGGTGCCATCTTCTGAAAAGGGT |
| SIPA1L2 | Signal-induced proliferation-associated 1 like 2 | TGTAAATGGCTCACCGAGTGGTCA | ACAAGTGGTTCCGCTGGCTCA |
| TCEAL2 | Transcription elongation factor A (SII)-like 2 | CTGTCTGAGCTGCCCAGGCG | CAACAGACGCGCAGACCTGC |
| TFF3 | Trefoil factor 3 (intestinal) | TGGGAGCTTGACAAAGGCATGCA | GCACCGTTGTTTGCACAGCTGC |
| TIMP1 | TIMP metallopeptidase inhibitor 1 | GACCTCGTCATCAGGGCCAAGTTC | CGGGGGTGTAGACGAACCGGA |
| TP53INP1 | Tumor protein p53 inducible nuclear protein 1 | AGCCCAAGTAGTCCCAGAGTGGA | TCCACTGGGAAGGGCGAAAGC |
| TRIB1 | Tribbles homolog 1 (Drosophila) | TGGACAGTGCTGAAATCAGGTGGT | ACGTGAGGTATACACGGCATTCTCA |
| TSPAN13 | Tetraspanin-13 | CCTGAACCAGGAGCAACAGGGTC | GCTAGCCAGACAGGTGTCATTTGGG |
| TSPAN15 | Tetraspanin 15 | CCTTGAGCCCTCTTGCAAGGGC | TGGGGGAAGGGCAAGAATAAGACA |
| VCAM1 | Vascular cell adhesion molecule 1 | TTCCACGCTGACCCTGAGCC | TCTAGGGAATGAGTAGAGCTCCACCT |
| ZNF783 | Zinc finger family member 783 | TGGGTAGAGAGGACCCTCGGG | GCTCCCATTGTCCTCTGCCGG |
